# Schistosomes contain divergent ligand-gated ion channels with an atypical Cys-loop motif

**DOI:** 10.17912/micropub.biology.000694

**Published:** 2023-01-11

**Authors:** Hailey Johnson, Mia VanHooreweghe, Jack A Satori, John D Chan

**Affiliations:** 1 University of Wisconsin - Oshkosh, Oshkosh, WI, USA

## Abstract

Ligand-gated ion channels (LGICs) are important regulators of neuromuscular function, making them attractive antiparasitic drug targets. While roundworm LGICs are targeted by several anthelmintic classes, flatworm LGICs are less studied. Chromosome-level genome assemblies have recently been released for *Schistosoma* flatworm species that cause the disease schistosomiasis. These have allowed us to comprehensively predict schistosome LGICs, adding to prior annotations. Analysis of LGIC sequences revealed a clade of receptors lacking cysteines at the eponymous Cys-loop region of the channel. Since these atypical channels are divergent from mammalian LGICs, they may be promising targets to treat diseases caused by parasitic flatworms.

**Figure 1. Schistosome ligand-gated ion channels include a clade of sequences missing cysteines within the Cys-loop:**
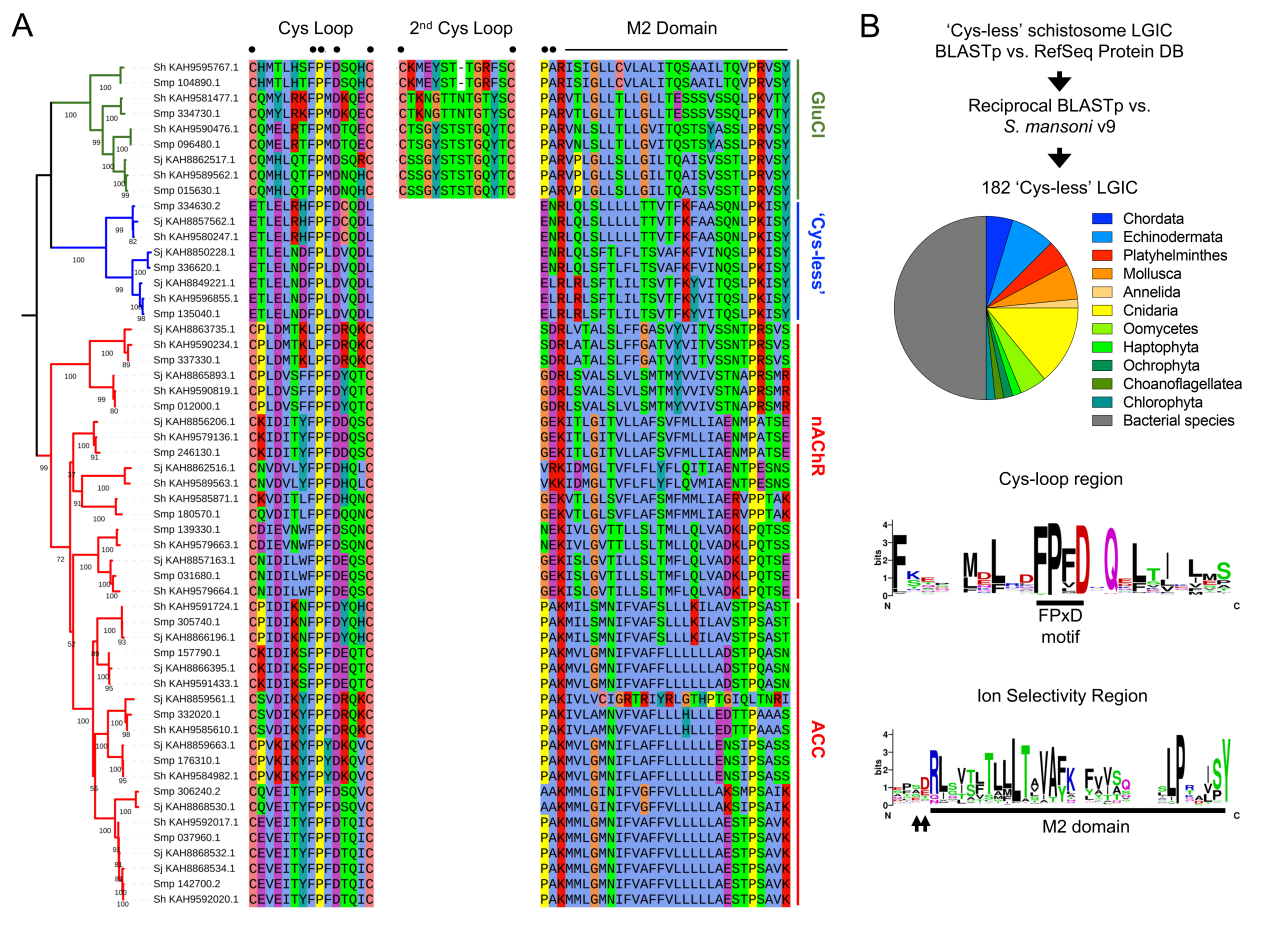
**(A)** Maximum likelihood tree of ligand-gated ion channels (LGICs) from *Schistosoma mansoni* (Smp), *Schistosoma japonicum* (Sj) and *Schistosoma haematobium* (Sh). Green clade = Glutamate-gated chloride channels (GluCls). Blue clade = ‘Cys-less’ LGICs. Red clade = Nicotinic acetylcholine receptors (nAchRs) and acetylcholine-gated chloride channels (ACCs). Amino acid sequences of Cys-loop and M2 domains shown to the right. Key positions in the alignment are noted with solid symbols (•). This includes cysteines flanking the two Cys-loops, the FPxD motif within the first Cys-loop, and the pore region just prior to the M2 domain which mediates ion selectivity. Partial sequences that did not contain either the Cys-loop or M2 region were omitted from the alignment shown in this figure, but are included in the fasta file of schistosome LGICs provided as extended data. **(B)** Search strategy to identify sequences similar to schistosome ‘Cys-less’ channels in other organisms. This search returned 182 sequences from the NCBI RefSeq Protein Database. Pie chart shows taxonomic distribution of species harboring these sequences. Plot of sequence conservation for these 182 hits is shown. Note the lack of cysteines at the beginning and end of the Cys-loop (which can be clearly identified by the conserved FPxD motif) and acidic amino acids prior to the M2 domain (arrowed).

## Description

Schistosomiasis is the neglected tropical disease caused by infection with *Schistosoma* parasitic flatworms. There are limited treatment options, with praziquantel the only therapy currently on the market. Alternative chemotherapies are needed since infections can be refractory to praziquantel treatment, raising the concern of potential drug resistance (Ismail et al. 1999; Melman et al. 2009; Kron et al. 2019).

Pentameric ligand-gated ion channels (LGICs) are targets of many antiparasitic drugs. For example, ivermectin targets glutamate-gated chloride channels and levamisole acts through nicotinic acetylcholine receptors to clear parasitic roundworms (Nixon et al. 2020). LGICs may also be effective targets for the development of antischistosomal therapies. Cholinergic disruption of parasite neuromuscular function has been well studied (Mellin et al. 1983; Day et al. 1996). The acetylcholinesterase inhibitor metrifonate is effective at treating human schistosomiasis (Bueding, Liu, and Rogers 1972; Jewsbury 1981), although its use has been discontinued in favor of praziquantel due to toxicity of organophosphates and lack of broad spectrum activity. It is plausible that acetylcholinesterase inhibitors work by increasing acetylcholine levels to non-selectively engage a milieu of cholinergic LGICs. Drugs that engage these LGICs more selectively may be safer and effective antischistosomal therapies. Numerous other classes of compounds which may be acting on LGICs exhibit *in vitro* or *in vivo* activity against schistosomes. These include benzodiazepines (Stohler 1978; McCusker et al. 2019), the avermectins, milbemycins, and spinosyns (Ryan et al. 2022), and the paralyzing effects of anesthetics (Khayyal 1965; Dickerson 1965).

Prior studies have cloned and functionally expressed several glutaminergic (Dufour et al. 2013) and cholinergic LGICs (MacDonald et al. 2014). Sequenced genomes for the major species of parasitic worms expanded the number of predicted LGICs in helminths (International Helminth Genomes Consortium 2019). Since then, chromosome level genome assemblies for all three schistosome species have recently become available (Buddenborg et al. 2021; Luo et al. 2022; Stroehlein et al. 2022), allowing us to comprehensively annotate schistosome LGICs.

Prior annotation of schistosome genomes had identified 14 S *chistosoma mansoni* LGICs, 14 *Schistosoma japonicum* LGICs and 19 *Schistosoma haematobium* LGICs (International Helminth Genomes Consortium 2019). *S. mansoni* gene IDs numbered 300000 and higher were not present in the genome release analyzed in (International Helminth Genomes Consortium 2019). We combined the flatworm datasets from this study and an annotation effort on a more recent *S. mansoni* genome (version 7) (McCusker et al. 2019). We used these sequences to build a HMMER profile for flatworm LGICs which was used to search recently released chromosome level schistosome genome assemblies (“HMMER” n.d.). Hit sequences were inspected for whether they possessed the four transmembrane domain architecture expected of LGICs and conserved motifs typical of these channels. In total, this analysis identified 21 *S. mansoni* LGICs, 22 *S. japonicum* LGICs and 20 *S. haematobium* LGICs. A maximum likelihood phylogenetic tree was generated which showed sequences clustering into clades corresponding to known glutamate-gated chloride channels (GluCls), an unusual clade of ‘Cys-less’ LGICs, nicotinic acetylcholine receptors (nAchRs) and acetylcholine-gated chloride channels (ACCs) ( **Figure 1** ). Previously described GluCls and ACCs contain an anion selectivity motif just prior to the M2 domain (ex. PAR or PAK) (Dufour et al. 2013; MacDonald et al. 2014). nAchRs generally possess amino acids known to confer cation selectivity (ex. GEK), but several sequences do contain amino acids other than the typical glutamic acid. Smp_337330 (SDR at this position) and Smp_012000 (GDR) both contain an aspartic acid instead of a glutamic acid, which may not impact ion selectivity given that they retain a negatively charged residue. However, Smp_321840 contains a positively charged lysine (VKK) at this position, which may well alter the ion selectivity of this channel.

Notably, eight sequences clustered into a distinct clade with no obvious relation to other annotated schistosome LGICs. These sequences are unusual in that they lack a pair of cysteines flanking the Cys-loop motif. Cysteines typically form a disulfide bond stabilizing the ligand-binding domain of the extracellular region of the channel. However, it is not entirely unprecedented for LGICs to lack cysteines at this position. Prokaryotes have diverse pentameric channels similar to eukaryotic LGICs and these also lack cysteines within the Cys-loop (Tasneem et al. 2005). Both prokaryotic and schistosome channels do still contain the conserved F/YPxD motif within the Cys-loop. Alphafold predictions of the protein structures for the schistosome sequences also indicate that the overall tertiary structure of these channels matches that expected of LGICs (ex. Uniprot A0A5K4FCR6, A0A5K4EMP5 and A0A5K4FB25) with beta sheets at the extracellular ligand binding domain followed by four alpha helices that comprise the M1 - M4 domains.

The ion selectivity region near the M2 domain is highly conserved in glutaminergic or cholinergic anionic (ex. PAK) or cationic receptors (ex. GEK). Schistosome ‘Cys-less’ receptors do not possess either of these ion selectivity motifs to allow obvious assignment as anion or cationic channels. However, all of the ‘Cys-less’ sequences do all contain a glutamic acid at the M2 -2’ position (amino acids near the M2 region are numbered according to (Keramidas et al. 2000)). This position is located at the cytoplasmic side of the channel pore. A negatively charged amino acid near this site is typical of cation channels. Ion selectivity of anionic glycine or GABA _C_ receptors is lost when the M2 -1’ position of these channels is experimentally mutated to a glutamic acid (Keramidas et al. 2000; Wotring, Miller, and Weiss 2003). Similarly, loss of glutamic acid at the nicotinic acetylcholine receptor M2 -1’ position contributes to a reversal of ion selectivity from cationic to anionic (Galzi et al. 1993). Other prokaryote ‘Cys-less’ ion channels, ELIC (Hilf and Dutzler 2008) and Glvi (Bocquet et al. 2007), are both selective for cations. Like the schistosome ‘Cys-less’ channels, both of these prokaryote channels contain a glutamic acid at the M2 -2’ position.

While uncommon, there are other reports of metazoan LGICs that do not contain cysteines in their Cys-loop. For example, one other ‘Cys-less’ receptor has been reported in the roundworm *Dirofilaria immitis* (Yates and Wolstenholme 2004). The *D. immitis* sequence (GenBank: CAE46431.1) is similar to *C. elegans lgc-34* , which also lacks cysteines within the Cys-loop motif, indicating ‘Cys-less’ channels are present among different clades of nematodes. This raises the question of whether schistosome ‘Cys-less’ channels may be similar to nematode ‘Cys-less’ channels. Comparison of the flatworm and roundworm ‘Cys-less’ LGICs reveals that they are actually quite different. The *D. immitis* sequence more closely resembles conventional anionic LGICs. While it lacks cysteines at the first Cys-loop, it does contain a second Cys-loop prior to the M1 domain, and that Cys-loop does contain two flanking cysteines. Presence of a second Cys-loop is characteristic of anionic LGICs such as glutamate and glycine receptors (Dent 2006), and schistosome ‘Cys-less’ sequences do not contain a second Cys-loop ( **Figure 1A** ). The ion selectivity of the *D. immitis* sequence is unclear, with a proline at the M2 -1’ position. As noted above, flatworm ‘Cys-less’ channels contain charged acid amino acids near this region more consistent with cation selectivity. Nematode ‘Cys-less’ channels may derive from a more conventional anionic LGIC ancestor which did originally contain cysteines in the Cys-loop motif, while schistosome channels may fall under a category of metazoan LGICs termed ‘Cys-less Pro-loop receptors’ that consists mainly of protist and lophotrochozoan sequences and has been described in (Jaiteh, Taly, and Hénin 2016). This study identified a clade of ‘Cys-less’ LGICs separate from typical metazoan anionic and cationic LGICs, although schistosome ‘Cys-less’ sequences were not included in this analysis, perhaps due to the incomplete state of schistosome genomes at that time.

We were interested in the taxonomic distribution of these schistosome ‘Cys-less’ channels, anticipating that they were members of the protist and lophotrochozoan ‘Cys-less’ clade reported in (Jaiteh, Taly, and Hénin 2016). A BLASTp search was performed against the NCBI RefSeq protein database using the Schistosome ‘Cys-less’ sequences. A reciprocal BLAST was then performed using these results as a query against the *S. mansoni* v9 proteome. All RefSeq protein sequences that returned either Smp_336620, Smp_334630 or Smp_135040 as a top hit were retained and aligned to confirm there were no cysteines in the Cys-loop. Organisms containing these channels covered a wide taxonomic distribution, including protostomes (Annelida, Mollusca, Platyhelminthes) and deuterostomes (Chordata, Echinodermata), as well as unicellular eukaryotes (Haptophyta, Monosiga, Micromonas, Oomycetes) and numerous bacterial phyla ( **Figure 1B** ). Often, these sequences were electronically annotated as glycine, GABAergic or cholinergic LGICs. In addition to lacking a pair of cysteines within the first Cys-loop, these sequences often contained an acidic amino acid at the M2 -1’ or -2’ position. It is possible that not all of the sequences identified in this search are orthologous. However, it does at the very least appear that sequences similar to schistosome ‘Cys-less’ LGICs appear across many phyla, especially considering that the proteomes in the RefSeq database are only a sampling across a diversity of taxonomic positions. No hits to schistosome ‘Cys-less’ channels were found in commonly studied model organisms such as humans, mice, *Drosophila* or *C. elegans* .

The function of schistosome ‘Cys-less’ LGICs is unknown. However, experiments on planarian flatworms may provide some insight. The free-living flatworm *Schmidtea mediterranea* contains LGICs that lack cysteines within the Cys-loop region and are homologs of schistosome ‘Cys-less’ LGICs. Two of these receptors, *gabrg1* and *gabrg2* , have been shown to be expressed in mechanosensory neurons (Ross et al. 2018). RNAi knockdown of these channels results in seizure-like movements and reduced sensory functions (Ross et al. 2018). Further work is required to determine the endogenous ligand and ion selectivity of these flatworm ‘Cys-less’ LGICs, which will allow for more precise nomenclature. Nevertheless, they are potentially attractive anthelmintic targets given their possible involvement in neuromuscular function and the fact that they are not found in humans.

## Methods

Flatworm LGIC sequences from (International Helminth Genomes Consortium 2019) and (McCusker et al. 2019) were used to create a flatworm LGIC HMMER profile which was used to search the genomes of *S. mansoni* (bioproject PRJEA36577), *S. japonicum* (bioproject PRJNA739049) and *S. haematobium* (bioproject PRJNA78265) using HMMER3 (version 3.3.2; http://hmmer.org/). For genes where there were multiple predicted transcripts, the longest open reading frame was chosen for inclusion in this analysis. Transmembrane domains were predicted using DeepTMHMM (version 1.0.18; Hallgren et al. 2022), and reciprocal BLAST searches were performed against a previous *S. mansoni* LGIC dataset (McCusker et al. 2019) to aid in annotation. Translated sequences were aligned using MUSCLE, and the alignment was trimmed using TrimAl at a 30% gap threshold (Capella-Gutiérrez, Silla-Martínez, and Gabaldón 2009). A maximum-likelihood phylogeny was generated using IQtree (version 2.2.0; 1000 bootstrap replicates) (Minh et al. 2020) and visualized using iTOL v6 (Letunic and Bork 2021). In order to to identify ‘Cys-less’ sequences across other phyla, the schistosome ’Cys-less’ receptors were used to perform a BLASTp search against the RefSeq Protein Database, and the results of this search were then used to perform a reciprocal BLAST against the *S. mansoni* v9 proteome. Sequences which matched to either either Smp_336620, Smp_334630 or Smp_135040 were retained and aligned with MUSCLE or MAFFT, and plots of conserved amino acids generated using WebLogo (Crooks et al. 2004).

## Extended Data

Description: Protein sequences of LGICs predicted in the genomes of S. mansoni, S. japonicum and S. haematobium.. Resource Type: Dataset. DOI: 10.22002/pkvds-7kg28
